# Prevalence of *Trypanosoma evansi* infection in Thai and imported beef cattle on the Thai-Myanmar border using parasitological and molecular methods

**DOI:** 10.14202/vetworld.2025.500-507

**Published:** 2025-02-27

**Authors:** Chanya Kengradomkij, Pairpailin Jhaiaun, Wissanuwat Chimnoi, Narisorn Piliean, Tawin Inpankaew, and Ketsarin Kamyingkird

**Affiliations:** 1 Department of Parasitology, Faculty of Veterinary Medicine, Kasetsart University, Lad Yao, Chatuchak, Bangkok, 10900, Thailand; 2 Department of Livestock Development, Sa Kaeo Animal Quarantine Station, Sa Kaeo Province, Thailand

**Keywords:** beef cattle, polymerase chain reaction, Thai-Myanmar border, *Trypanosoma evansi*, trypanosomiasis, vector-borne diseases

## Abstract

**Background and Aim:**

Animal trypanosomiasis caused by *Trypanosoma evansi* is a major vector-borne disease affecting livestock productivity, especially in tropical regions. The disease has been documented in Thailand in cattle, buffaloes, and other livestock. This study aimed to estimate the prevalence of *T. evansi* infections in Thai and imported beef cattle along the Thai-Myanmar border using parasitological and molecular diagnostic methods.

**Materials and Methods:**

A cross-sectional study was conducted on 347 cattle, comprising 120 Thai cattle and 227 imported cattle, during December 2022 and January 2023. Blood samples were collected and tested using hematocrit centrifugation technique (HCT), buffy coat smear microscopic examination (BMIC), and polymerase chain reaction (PCR) targeting the *internal transcribed spacer-2* region. Statistical analysis was performed to assess the sensitivity and specificity of diagnostic methods and evaluate risk factors such as sex, age, and breed.

**Results:**

Trypanosomes were detected in 2.59% of samples using HCT and 0.58% using BMIC, while *T. evansi* was confirmed in 2.02% of samples using PCR. Among Thai cattle, *T. evansi* prevalence was 2.5%, compared to 1.8% in imported cattle (p = 0.64). Male cattle showed significantly higher infection rates (3.2%) compared to females (0%, p = 0.04). Younger cattle (<2 years) exhibited slightly higher prevalence than older groups. Sensitivity and specificity of HCT and BMIC were 14.28% and 97.65%, and 14.28% and 99.70%, respectively, compared to PCR.

**Conclusion:**

The study highlights the importance of routine laboratory diagnostics, particularly PCR, to confirm *T. evansi* infections in livestock, especially in high-risk areas like the Thai-Myanmar border. Findings emphasize the need for integrated diagnostic approaches to improve detection and control measures. Collaborative efforts between government agencies and veterinary experts are recommended to manage trypanosomiasis and reduce its impact on livestock productivity and livelihoods.

## INTRODUCTION

*Trypanosoma evansi* is a hemoflagellate protozoan parasite that causes trypanosomiasis, also known as surra. It is highly pathogenic in camelids and equids, but it also affects a wide range of domestic and wild animals, including cattle, buffaloes, sheep, goats, pigs, dogs, deer, gazelles, and elephants [1, 2]. *T. evansi* has a broad geographic distribution, being widely prevalent in regions such as North Africa, the Middle East, South America, and Asia [1–5]. Biting flies, including *Tabanus*, *Stomoxys*, and *Haematopota*, have been identified as mechanical vectors for *T. evansi* [1, 6–8]. Infected animals exhibit clinical signs such as progressive emaciation, anemia, edema, pyrexia, and weight loss, and in severe cases, death [2, 9].

Detection of *T. evansi* infection can be performed using parasitological (hematocrit centrifugation technique [HCT], microscopic examination, and buffy coat smear microscopic examination [BMIC]), serological (agglutination test and enzyme-linked immunosorbent assay), molecular techniques (polymerase chain reaction [PCR] and nested PCR), and bioassay (mouse inoculation test). However, limitations of the current diagnostic methods to confirm *T. evansi* infection, such as HCT, are fast and easy to detect live trypanosomes but cannot confirm trypanosome species. BMIC is cheap and can be used to confirm trypanosome species but has low sensitivity, and PCR is specific and sensitive for confirming trypanosome species but costly and time-consuming. Therefore, multiple diagnostic techniques are recommended to confirm *T. evansi* infection. In Thailand, *T. evansi* infection has been documented in cattle [6], water buffaloes [10, 11], elephants [12], horses [13], and dogs [14]. Trypanosomiasis causes abortions in cattle and buffaloes [15], leading to significant economic losses due to reduced productivity and high treatment costs [3, 10]. The reported prevalence of *T. evansi* in Thailand is 8.1% in dairy cows [16], 7.66% in beef cattle [17], and between 5.23% and 12.2% in buffaloes [7, 11]. Variations in prevalence rates are influenced by factors such as detection method, geographic location, farm management practices, herd size, breeding strategies, and the sex of the animals [17].

*T. evansi* infection in cattle lowers milk and meat production, leading to lowering farmers’ income and increasing the cost of treatment and control. This is one of the important vector-borne diseases that require insect pest management and the use of insecticides, which directly impact the environment and health issues. Therefore, this neglected trypanosomiasis remains a problem for livestock farmers and all levels of livestock production areas, including the Thai and Thai-Myanmar border, where people raise cattle as one of their food sources and impact on socioeconomic and ecology. Thailand shares its borders with Myanmar to the northwest, Laos to the northeast and east, and Cambodia to the southeast [18]. Animal products are transported from neighboring countries to Thailand through terrestrial borders. Kanchanaburi Province, located in western Thailand and bordering Myanmar, is a key area for such transit. The animal quarantine station (AQS) in the Sangkhla Buri district of Kanchanaburi oversees the inspection and quarantine of live animals and animal products entering Thailand. The quarantine process is critical for preventing the spread of infectious and emerging diseases. Consequently, Kanchanaburi Province is considered a high-risk area for diseases such as trypanosomiasis, which can be transmitted through reservoir animals and biting insect vectors.

While numerous studies have investigated *T. evansi* infections in animals within Thailand, there is a notable lack of research focusing on imported cattle entering through the Thai-Myanmar border. Therefore, the objectives of this study are as follows: (1) to detect *T. evansi* infection in Thai beef cattle and imported beef cattle from the Thai-Myanmar border using parasitological and PCR methods, and (2) to examine potential risk factors associated with *T. evansi* infection.

## MATERIALS AND METHODS

### Ethical approval

All samples were collected from livestock in accordance with the animal use protocol, which was reviewed and approved by the Kasetsart University Institutional Animal Care and Use Committee (Approval number ACKU65-VET-095).

### Study period and location

**Figure 1:**
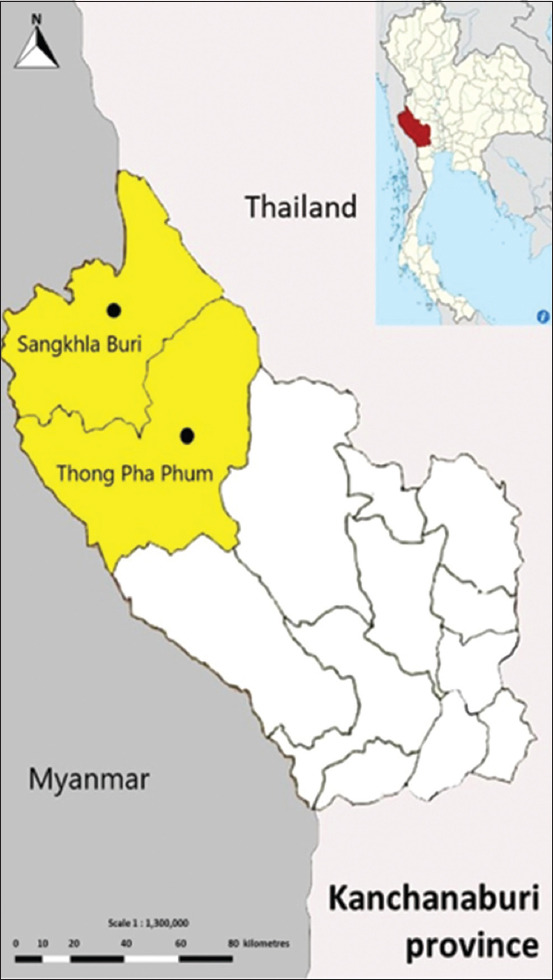
Geographical areas of *Trypanosoma evansi* infection in cattle in this study [Source: https://commons.wikimedia.org/wiki/User: NordNordWest].

The sampling was conducted during December 2022 and January 2023. Kanchanaburi is a province in western Thailand that shares a border with Myanmar. The province comprises 13 districts, four of which are adjacent to neighboring areas. The study area was selected based on farms located in the Sangkhla Buri and Thong Pha Phum districts, as well as the AQS (Figure 1).

### Sample collection

The sample size was calculated using the average estimated prevalence (p) of *T. evansi* among cattle in Thailand, a 95% confidence level (t), and a 5% margin of error (m), as follows: n = t^2^ × p (1 − p)/m^2^. The minimum sample size was estimated to be 112.

Criteria for cattle sampling include the position of the farm near Thai-Myanmar border, cattle raising on a small-scale farm, cattle older than 6 months with no pregnancies, and those restrained by the owner. Blood samples were collected from the jugular vein and stored in ethylenediaminetetraacetic acid tubes for parasitological identification and deoxyribonucleic acid (DNA) extraction. A total of 347 samples were obtained, including 120 from Thai cattle and 277 from imported cattle. Data collection factors included sex, age, and breed.

### Parasitological identification of *T. evansi*

#### HCT or Woo’s technique

Whole blood samples were placed in a hematocrit tube with one end sealed using plasticine wax and centrifuged at 11,200× *g* for 5 min. Following centrifugation, the plasma-buffy coat interface was examined under a light microscope (Olympus CX23, Japan) to detect trypanosome movement. Observations were made at the appropriate depth through the capillary tube [19]. The pack cell volume (PCV) was measured using a hematocrit capillary tube reader [20].

#### BMIC

After recording the PCV, the hematocrit tube was cut from the buffy coat using a diamond pen. A thin smear of the buffy coat was prepared, air-dried, fixed with absolute methanol, and stained using a modified Wright Giemsa. The identification of trypanosomes was conducted through microscopic examination of the stained buffy-coat smear [19].

### Molecular detection of *T. evansi* using PCR

#### DNA extraction

Genomic DNA was extracted from 200 μL of whole blood using a NucleoSpin® Blood DNA extraction kit (MACHEREY-NAGEL, Germany). The final DNA elution volume was reduced to 100 μL and stored at −20°C until further use.

#### Conventional PCR targeting the trypanozoon internal transcribed spacer-2 (ITS 2) gene

The PCR assay was performed using the primers ITS2F (5’-TGTCACGCATATACGTGTGTG-3’) and ITS2R (5’-TACACACATACACACTATCCG-3’), which target the *ITS2* regions that are divergent among species in the subgenus Trypanozoon [21]. Amplification was carried out in 20 μL reaction mixture containing PCR buffer (New England Biolabs, USA), 50 mM MgCl_2_, 10 mM dNTPs mix, 10 μM of each primer, 0.5 units of Taq DNA polymerase, and 2 μL of DNA template. Positive control (DNA of *T. evansi*) and negative control (distilled water) samples were included in the assay. Thermal cycling conditions consisted of an initial denaturation step at 94°C for 1 min, followed by 30 cycles of denaturation at 94°C for 30 s, annealing at 60°C for 1 min, and extension at 72°C for 30 s, with a final extension step at 72°C for 2 min. PCR products were stained and analyzed by electrophoresis through a 1.5% agarose gel. A 347-bp product was identified as positive under an ultraviolet transilluminator (Syngene Bio Imaging, United Kingdom).

### Statistical analysis

The statistical analysis was conducted to evaluate the diagnostic performance of HCT and BMIC compared to PCR as the reference standard. Sensitivity, specificity, positive predictive value, and negative predictive value were calculated for each diagnostic method using a 2 × 2 contingency table, and 95% confidence intervals were reported to estimate the precision of these metrics.

Descriptive statistics were used to summarize the characteristics of the study population, including means and standard deviations for continuous variables such as PCV levels and proportions for categorical variables such as sex, breed, and infection prevalence. Differences in infection prevalence between Thai and imported cattle were analyzed using the Chi-square test, or Fisher’s exact test where appropriate. For continuous variables like PCV levels, independent t-tests or Mann-Whitney U-tests were applied based on the distribution of data, which was tested using the Shapiro-Wilk test for normality.

Risk factor analysis was performed using logistic regression to assess the association between *T. evansi* infection and independent variables such as sex, age, breed, and PCV levels. Univariate logistic regression was initially used to identify significant associations, and variables with p < 0.2 were included in a multivariable logistic regression model. Odds ratios with 95% confidence intervals were calculated to quantify the strength of associations. Multicollinearity among independent variables was evaluated using variance inflation factors.

All analyses were conducted using appropriate statistical software, and statistical significance was determined at p < 0.05. Results are presented as summary tables and supported by visual aids to enhance interpretation. Limitations such as sample size constraints and lack of seasonal data were acknowledged to provide context for the findings.

## RESULTS

**Figure 2:**
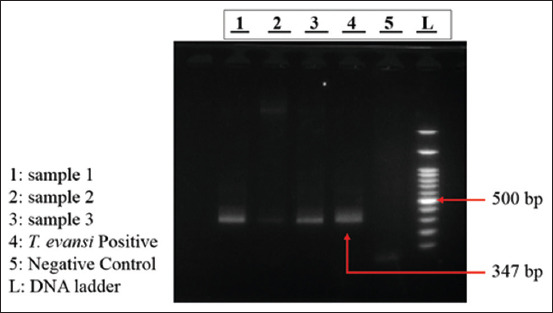
Amplification of the *Trypanosoma evansi* *ITS2* gene in agarose gel electrophoresis.

Parasitological detection revealed that trypanosomes were identified in 9/347 animals (2.59%) using the HCT method and in 2/347 animals (0.58%) using the BMIC method (Table 1). PCR analysis demonstrated that *T. evansi* infections were detected in 7/347 animals (2.02%) (Figure 2 and Table 1). Among Thai cattle, *T. evansi* was detected in 3/120 cattle (2.5%), whereas 4/227 imported cattle (1.8%) tested positive (Table 1). The discrepancy of each diagnostic test revealed that the HCT method could detect all live trypanosomes in the freshly collected blood, whereas BMIC may lose its sensitivity due to the low number of parasites. The comparison between HCT and PCR methods revealed that not all live trypanosomes found in HCT were confirmed as *T. evansi* using PCR. This may be due to different trypanosome species, such as *Trypanosoma theileri*, that may also be found in infected cattle. A comparison of the sensitivity and specificity of the HCT and BMIC methods for trypanosome detection showed 14.28%, 97.65%, and 14.28%, 99.70%, respectively.

**Table 1:** Prevalence of *Trypanosoma evansi* in beef cattle using HCT, BMIC, and molecular methods.

Animal population	Tested animals	Positive using the HCT method (%)	Positive using the BMIC method (%)	Positive using PCR (%)
Thai cattle	120	9 (7.50)	2 (1.67)	3 (2.50)
Imported cattle	227	0 (0)	0 (0)	4 (1.76)
Total	347	9 (2.59)	2 (0.58)	7 (2.02)

HCT=Hematocrit centrifugation technique, BMIC=Buffy coat smear microscopic examination, PCR=Polymerase chain reaction

Risk factor analysis indicated statistically significant differences in *T. evansi* infection rates between male and female cattle (p = 0.04) (Table 2). Specifically, *T. evansi* infections were found in 7/217 males (3.2%) but were absent in females. Age group analysis revealed higher *T. evansi* infection rates in younger cattle (<2 years) compared to older groups (2–5 years and >5 years). Local breeds exhibited more infections than mixed breeds (Table 2).

**Table 2:** Risk factors associated with *T. evansi* infection in Thai and Thai-Myanmar border cattle.

Factors	Parameters	*T. evansi* PCR-positive/ tested (%)	Statistical parameters
Animal groups	Thai cattle	3/120 (2.5)	χ^2^ = 0.22, df = 1, p = 0.64
	Imported cattle	4/227 (1.8)	
Sex	Male	7/217 (3.2)	χ^2^ = 4.28, df = 1, p = 0.04
	Female	0/130 (0)	
Age groups	<2 years	1/26 (3.8)	χ^2^ = 0.81, df = 2, p = 0.67
	2–5 years	6/303 (2.0)	
	>5 years	0/18 (0)	
Breeds	Native breed	6/288 (2.1)	χ^2^ = 0.04, df = 1, and p = 0.85.
	Mixed breed	1/59 (1.7)	
PCV groups	>24%	7/339 (2.1)	χ^2^ = 0.13, df = 1, p = 0.72
	<24%	0/6 (0)	

*T. evansi*=*Trypanosoma evansi*, df=Degree of freedom, PCR=Polymerase chain reaction, PCV=Pack cell volume

PCV values ranged from 20.5% to 49% among all cattle, with no significant difference observed in *T. evansi* infection rates between animals with normal PCV values and those in the anemic group (PCV% lower than 24) (Table 2). The average PCV among *T. evansi-*infected cattle was 31.93% (range: 25%–39%) compared with 34.73% (range: 20.5%–49%) in uninfected cattle. In addition, there was no statistically significant difference in *T. evansi* infection rates between Thai and imported cattle (p = 0.64) (Table 2).

## DISCUSSION

This study demonstrated that the HCT method detected more trypanosomes than the BMIC method, consistent with findings from previous studies conducted in Khon Kaen, Chiang Mai, and Lum Pun [17, 22]. Approximately 70–100 μL of whole blood is generally used for HCT preparation, whereas 3–5 μL of the buffy coat is used for thin blood smear preparation in the BMIC method [19]. The higher sensitivity of the HCT method compared with BMIC may be attributed to the potential loss of parasites during the preparation of thin blood smears, especially in samples with low parasitemia.

PCR is recognized as a highly specific and sensitive method compared with parasitological and serological approaches [23], with the ability to detect at least 1 trypanosome per ml of blood [24]. Thus, PCR is suitable for screening animals infected with *T. evansi*. In this study, PCR results confirmed *T. evansi* infection in 1.76% of imported cattle and 2.50% of Thai cattle. In addition, while nine cattle tested positive for trypanosomes using the HCT method, only two samples were morphologically identified as *T. evansi* and *T. theileri*. PCR results revealed a *T. evansi* infection rate of 2.02% (7 samples), but only one sample tested positive by both PCR and HCT methods, confirming it as *T. evansi*. The remaining six HCT-positive samples, which were PCR-negative, might belong to *T. theileri*, necessitating further confirmation. The findings from this study indicate that PCR is significantly more specific and less sensitive than other parasitological identification methods due to its species-level specificity. Therefore, the sensitivity of the HCT and BMIC methods is lower when PCR is used as the standard diagnostic method. Routine diagnostic methods using HCT or BMIC should be confirmed by PCR for further animal health management and disease surveillance. Moreover, to increase the impact of *T. evansi* diagnosis for better treatment and control of infected cases, field applications using *T. evansi* detection tests that are fast and easy to use in the field, such as card agglutination tests, immunochromatographic tests, and recombinase polymerase amplification lateral flow assays, should be developed and applied.

The molecular prevalence of *T. evansi* infections in cattle has been reported globally [4, 25–30]. In Thai cattle, a previous study recorded a lower prevalence of *T. evansi* infections at 0.9% [31], while another report indicated higher infection rates ranging from 29.5% to 34.3% [32, 33]. The variation in infection rates may result from differences in the primers used, the age and breed of the animals, geographical sampling locations, and the abundance of vectors [31, 32, 34]. This study analyzed samples from both Thai and imported cattle, finding a slightly higher infection rate in Thai cattle than in imported cattle near the Thai-Myanmar border; however, the difference was not statistically significant. However, the higher *T. evansi* prevalence in Thai cattle may reflect the lack of prevention and control of biting insects that are responsible for *T. evansi* transmission as well as the lack of treatment of reservoir cattle in this area. Hence, imported cattle should be treated and quarantined before moving or being transported to the border, which affects the prevalence of *T. evansi* to a lower level than Thai cattle. These findings suggest that *T. evansi* is widespread in both Thai and imported cattle, potentially due to shared habitats of hematophagous insect abundance that transmit the pathogen to the border region.

The risk factor analysis indicated that sex was associated with *T. evansi* infection. Male cattle were found to have a significantly higher prevalence of infection compared to females (p = 0.04), a finding consistent with previous studies in buffaloes [10] and cattle [29, 35, 36]. Abera *et al*. [36] explained that male animals are often used for drought or transportation, which increases their exposure to biting insects. The results of the present study showed a higher prevalence in males, indicating that males have more chances of being exposed to biting insects than females.

The higher prevalence in younger animals may be due to their longer resting periods compared with older animals [37], which increases the likelihood of insect biting. In addition, this was probably due to the relationship between animal age and age-acquired immunity, including trypanocidal treatments, which are more frequently administered to adults [38].

Native breeds exhibited higher infection rates than mixed breeds, suggesting that native cattle are more susceptible to *T. evansi*. However, no statistically significant difference in infection rates was observed between the two breeds in this study.

The PCV values in this study ranged from 20.5% to 49%. Anemic cattle (PCV <24%) did not show an association with *T. evansi* infection (p = 0.72), consistent with findings from a previous study conducted in eastern Thailand [39]. The average PCV in *T. evansi*-infected cattle (PCV = 31.93%) was slightly lower than that of the uninfected cattle (34.73%). Despite this, other clinical signs should be considered when evaluating infection.

Cattle generally remain asymptomatic and act as reservoirs for infectious agents [28]. Although the prevalence of *T. evansi* infections investigated in this study was low, it remains a potential risk. Accurate diagnostic tests are crucial for controlling trypanosomiasis, enabling a better understanding of epidemiology and facilitating effective treatment and prevention strategies. Animal trypanosomiasis caused by *T. evansi* infection is highly endemic throughout Thailand and Southeast Asia. Live animal transportation is an important disease-spreading route for vector-borne parasitic diseases. A sensitive test during animal quarantine throughout Southeast Asia is recommended.

## CONCLUSION

This study highlights the importance of accurate and routine diagnostic screening for *T. evansi* infections in cattle along the Thai-Myanmar border. Using parasitological techniques (HCT and BMIC) and molecular methods (PCR), the study identified a *T. evansi* infection prevalence of 2.02%, with slightly higher rates in Thai cattle (2.5%) compared to imported cattle (1.8%), although the difference was not statistically significant. Male cattle were found to be at higher risk of infection (p = 0.04), while younger animals also showed a marginally increased prevalence. HCT demonstrated a higher ability to detect live trypanosomes compared to BMIC, but PCR proved to be the most sensitive and specific method for species confirmation.

The strength of this study lies in its application of multiple diagnostic approaches, providing comprehensive insights into the limitations and advantages of each technique. The inclusion of both Thai and imported cattle further adds value by addressing a significant knowledge gap regarding cross-border livestock health.

However, the study is limited by a relatively small sample size, absence of seasonal sampling, and reliance on a single primer set in PCR for species identification. These factors may have influenced the overall prevalence estimates and reduced the ability to explore the full diversity of trypanosome species.

Future studies should focus on larger sample sizes and incorporate seasonal variability to provide a more representative understanding of infection dynamics. In addition, advanced molecular techniques such as whole-genome sequencing or multiplex PCR could be employed to differentiate co-infections and further enhance diagnostic accuracy. Implementation of field-friendly diagnostic tools, such as immunochromatographic tests, could improve surveillance and control measures in endemic regions. Cross-border collaborations and strict quarantine protocols are recommended to mitigate the risk of disease transmission through livestock movement.

Integrating molecular diagnostics into routine livestock health management systems and prioritizing research on sustainable vector control strategies will be critical to reducing the burden of *T. evansi* and safeguarding livestock productivity in the region.
